# Effect of Continuous Positive Airway Pressure (CPAP) Mode on Lung Function, Exercise Tolerance, Vital Signs, and Dyspnea After Acute SARS-CoV-2 Infection †

**DOI:** 10.3390/clinpract15040073

**Published:** 2025-04-02

**Authors:** Emilia Raposo Nascimento, Paloma Lopes Francisco Parazzi, Fernando Augusto Lima Marson, Maria Ângela Gonçalves Oliveira Ribeiro, Carla Cristina Sousa Gomez, Patrícia Blau Margosian Conti, Bianca Aparecida Siqueira, Edvane Aparecida Braz Araújo Silva, José Dirceu Ribeiro

**Affiliations:** 1 Center for Research in Pediatrics, University of Campinas, Campinas 13083-871, SP, Brazil; ernemilia@unicamp.br (E.R.N.); palomafp@unicamp.br (P.L.F.P.); or fernandolimamarson@hotmail.com (F.A.L.M.); angelagr@unicamp.br (M.Â.G.O.R.); carlacg@unicamp.br (C.C.S.G.); patriciablau@unicamp.br (P.B.M.C.); 2 Laboratory of Molecular Biology and Genetics, Laboratory of Clinical and Molecular Microbiology, LunGuardian Research Group—Epidemiology of Respiratory and Infectious Diseases, Postgraduate Program in Health Science, São Francisco University, Bragança Paulista 12916-900, SP, Brazil; bianca.siqueira@mail.usf.edu.br; 3 Collaborating Physiotherapist, São João da Boa Vista 13876-750, SP, Brazil; edvanearaujo@unicamp.br

**Keywords:** Borg scale, coronavirus disease (COVID-19), heart rate, non-invasive ventilation, pandemic, peripheral oxygen saturation, physical activity, respiratory rate, severe acute respiratory syndrome coronavirus 2

## Abstract

**Background/Objectives:** The coronavirus disease (COVID-19) pandemic was associated with an intense impact on health worldwide. Among the sequelae, it became necessary to clarify respiratory impairment related to lung function and aerobic capacity, as well as the treatment of curative and preventive measures of pulmonary involvement. In this context, this study aimed to compare vital signs, the sensation of dyspnea (Borg scale), lung function, and exercise tolerance before and after the use of non-invasive mechanical ventilation (NIV) in adults of both sexes after acute infection with severe acute respiratory syndrome coronavirus 2 (SARS-CoV-2). **Methods:** A cross-sectional analytical clinical study was performed with the inclusion of individuals who had been diagnosed with COVID-19 at least three months before data collection. Individuals were evaluated for vital signs (heart rate and peripheral oxygen saturation), Borg scale, spirometry, and submaximal exercise protocol of two minutes of the step test before and after receiving NIV in ventilation mode by continuous positive airway pressure of 6 cm H_2_O for 30 min. **Results:** A total of 50 participants were enrolled and grouped as a mild (N = 25) or severe (N = 25) clinical phenotype during SARS-CoV-2 infection according to the criteria of the World Health Organization. In our data, the forced vital capacity (*p* < 0.001), the ratio between the forced expiratory volume in the first one second to the forced vital capacity and the forced vital capacity (*p* = 0.020), and the two-minute submaximal step exercise protocol (number of steps—*p* = 0.001) showed a statistical improvement in the severe clinical phenotype group after NIV. In addition, forced expiratory volume in the first one second to the forced vital capacity (*p* = 0.032) and the two-minute submaximal step exercise protocol (number of steps—*p* < 0.001) showed a statistical improvement in the mild clinical phenotype group after NIV. No changes were described for vital signs and the Borg scale. **Conclusions:** This study allowed us to identify that NIV is a tool that promotes better exercise capacity by increasing the number of steps achieved in both clinical phenotype groups and improving lung function observed in the spirometry markers.

## 1. Introduction

The coronavirus disease (COVID-19) caused a great impact worldwide during recent years and, even after the pandemic peak, the human population has experienced problems associated with outcomes arising from viral infection, including long-lasting symptoms considered to be post-COVID-19 syndrome [1,2,3]. Post-COVID-19 syndrome includes a multiplicity of complaints, symptoms, or sequelae that arise after acute infection with severe acute respiratory syndrome coronavirus 2 (SARS-CoV-2) [3,4,5]. Due to the heterogeneity of phenotypes, there is no single terminology to define post-COVID-19; however, it is variably defined as the persistence of symptoms or sequelae that occur at least between the fourth week after SARS-CoV-2 infection that remains long after (beyond 12 weeks) [6,7].

The symptoms reported in post-COVID-19 syndrome are generally related to reduced lung function, impaired ability to work and care for themselves, low quality of life, and high consumption of healthcare [3,4,6,7]. In the literature, the five most common symptoms include fatigue, memory deficit, sleep problems, joint pain, and dyspnea [4,6]. Dyspnea, as well as thromboembolism, are the most important sequelae present in patients who have post-COVID-19 [8]. Dyspnea is a predictor of morbidity and mortality in the general population and is associated with reduced functional capacity and quality of life, which can result in refusal, discontinuity, and the inability to carry out daily activities [9,10,11].

Although the cause–effect relationship between viral infections and decreased lung function can be difficult to establish, there is evidence to suggest that some episodes of viral infections contribute to a long-term decline in lung function [12,13]. Regarding the COVID-19 pandemic, it was suggested that a reduction in lung function is due to SARS-CoV-2 infection in the medium term and long term [13,14]. COVID-19 can cause extensive injury to alveolar epithelial cells and endothelial cells with secondary fibroproliferation, indicating the potential for chronic vascular and alveolar remodeling leading to pulmonary fibrosis and pulmonary hypertension [14,15]. Patients with COVID-19 presented pneumomediastinum and injured trachea and airways due to an abnormal regenerative process with cartilaginous tissue remodeling. For example, a study described the presence of fibrohyaline degeneration of the tracheal rings found in patients with COVID-19 [16]. In addition, it was not possible to establish when irreversible post-COVID-19 pulmonary fibrosis occurs, as survivors of COVID-19, mainly severe cases, can show functional and tomographic improvement at follow-up [15].

Tomographic findings such as the presence of a dispersed bilateral distribution of lesions, a higher number of involved lobes, consolidations, and bronchial distortion, as well as the absence of mixed and reticular patterns, are associated with a poor prognosis in patients admitted to the intensive care unit. However, there is still no standardization of the tomographic findings and predictors of long-term morbidity [17]. Individuals infected with SARS-CoV-2, especially cases classified as severe and of a longer duration, may have chronic lung lesions with architectural distortion and residual abnormalities, as seen on computed tomography, functional impairment, and reduced exercise capacity in the long term [15]. Although definitive diagnosis depends on the reverse transcriptase-polymerase chain reaction in real-time [16], chest computed tomography is a valuable modality to measure the extent of lung involvement and propose a treatment plan. Prolonged lung involvement from COVID-19 pneumonia can be predicted in clinics from the patient’s initial symptoms, vital signs, and laboratory tests, including the presence of anosmia and low peripheral oxygen saturation, as well as elevated respiratory rate, white blood cell count, and c-reactive protein. Therefore, these patients should be considered high-risk patients for further medical planning [18].

The use of non-invasive mechanical ventilation (NIV) and its association with physical exercise can improve oxygenation, decrease dyspnea, and increase the distance covered by individuals in submaximal effort tests [19,20,21]. NIV can reduce the work of breathing and increase physical performance by increasing oxygenation in peripheral muscle microcirculation, improving local blood flow, and increasing transpulmonary pressure, facilitating alveolar ventilation [22,23]. Therefore, NIV could delay muscle fatigue by changing arterial blood flow from respiratory muscles to lower extremities muscles [21]. Inspiratory muscle fatigue can limit physical performance, leading to muscle metaboreflex activation that reduces blood flow to active skeletal muscles and exacerbates peripheral muscle fatigue [24,25]. In brief, when the work of breathing is reduced during NIV, a proportional increase in limb blood flow and vascular conductance can occur in the organism [19,20,21].

Individuals who have had a COVID-19 diagnosis can show a restrictive pulmonary pattern from hospital discharge to a prolonged and undefined time after the disease, which can reduce their quality of life and capacity to perform exercises and also increase their risk of comorbidities and death [3,13,25,26,27]. The assessment of physical capacity in individuals with chronic lung diseases or lung sequelae, such as those described in post-COVID-19 syndrome, can be carried out using incremental tests that measure gas exchange and cardiorespiratory parameters that limit effort and determine training load. The assessment of physical capacity must be carried out using a maximal or submaximal effort test. In this context, the study of strategies aimed at minimizing cardiorespiratory impairment during physical exercise has been a challenge for the scientific community [27,28]. There is evidence that NIV helps, during physical exercise, to improve lung function in people with chronic lung disease, promoting better physical performance and improvement in respiratory function. The evidence is based on the fact that NIV favors, in addition to lung function and respiratory muscles, increased resistance to muscular fatigue and reduced lower limb impairment. However, there is still little scientific evidence on the use of NIV during physical exercise in respiratory and cardiovascular biomarkers in individuals affected by COVID-19 or with sequelae resulting from SARS-CoV-2 infection [29].

In this context, individuals infected with SARS-CoV-2, mainly those with severe phenotypes, could benefit from NIV therapy after hospital discharge. Therefore, our study aimed to compare vital signs (heart rate and peripheral oxygen saturation), the sensation of dyspnea (Borg scale), lung function (spirometry), and exercise tolerance (two-minute submaximal step exercise protocol) before and after receiving NIV in adults after acute SARS-CoV-2 infection (post-COVID-19 syndrome) who had severe or mild phenotypes of COVID-19 during active infection.

## 2. Methods

### 2.1. Ethical Aspects

This study was approved by the Research Ethics Committee of the University of Campinas (no. 46403921.3.0000.5404). This study was conducted in accordance with the principles of the Declaration of Helsinki and all patients provided informed consent prior to enrollment.

### 2.2. Study Population and Protocols Used in This Study to Measure Biomarkers

**Figure 1:**
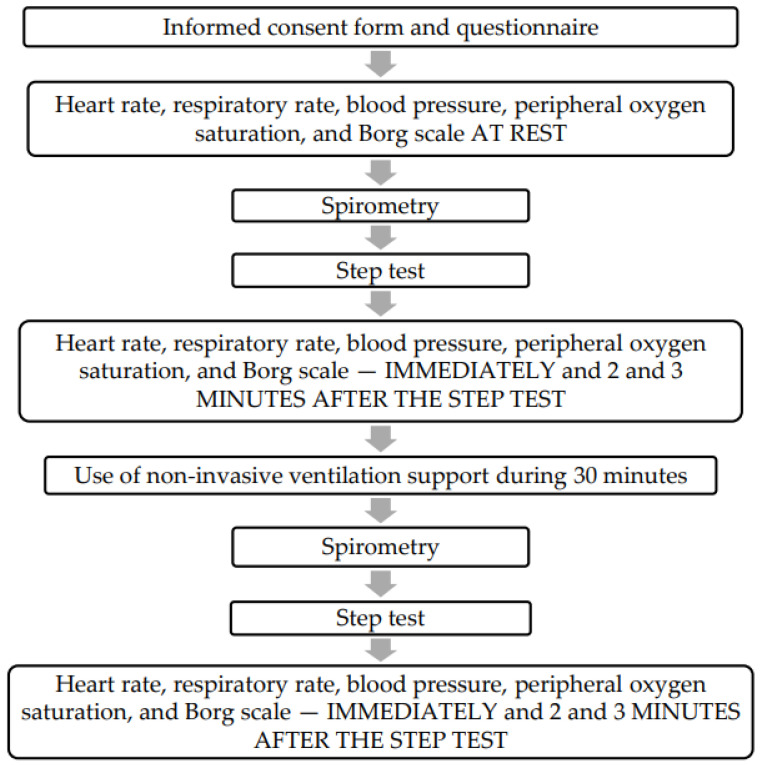
Summary of the study protocol.

A cross-sectional analytical clinical study was conducted with 50 individuals between 18 and 70 years of age of both sexes. All individuals were diagnosed with COVID-19 during hospitalization and were enrolled after hospital discharge. Data collection was carried out in 2022 at least 3 months and up to 12 months after hospital discharge due to acute SARS-CoV-2 infection. In our study, we used a convenience sample recruited for one year. Furthermore, all biomarkers were evaluated by trained healthcare professionals according to local guidelines and recommendations. The data collection was performed at home by the team staff that accommodated all equipment and instruments in the place where the participants were evaluated. The data collection was performed at home to increase the adhesion and to reduce the chance of a new infection because the center used in this study was a reference center to manage patients with a severe COVID-19 phenotype during the COVID-19 pandemic in Brazil. In brief, the summary of the study protocol is presented in Figure 1.

Participants were grouped according to the severity of COVID-19 into two groups following the criteria of the World Health Organization [30,31]:

Severe phenotype (N = 25 participants): presence of respiratory distress syndrome, septic shock, or other conditions that required mechanical ventilation therapy, whether invasive or NIV, during hospitalization.

Mild phenotype (N = 25 participants): absence of severe or critical symptoms and absence of ventilatory support or oxygen supplementation during hospital consultation.

For both groups, medical records were evaluated to collect information on comorbidities (presence or absence), pulmonary involvement on computed tomography (%; classified as <50% or ≥50%), adverse events such as pneumothorax and/or pneumomediastinum, vaccination status against COVID-19 during active SARS-CoV-2 infection (one or two doses), days of hospitalization (when needed), days of NIV use (when needed) during hospitalization, and type of positive pressure interface used (when needed) during hospitalization. The pulmonary involvement in computed tomography was evaluated by two trained medical doctors. The medical records were evaluated by the researchers of the study and validated by at least two authors. In cases of discordance, a third author was consulted. Additionally, the personal contact of the participants of the study was obtained in the medical records after authorization of the Research Ethics Committee of the University of Campinas.

The COVID-19 diagnosis was carried out based on clinical and epidemiological data associated with the positive real-time polymerase chain reaction test, by collecting nasal and oropharyngeal smears and/or by serological examination [Immunoglobulin G (IgG), Immunoglobulin A (IgA), and Immunoglobulin M (IgM)] [3,30,31,32].

Seven days before applying the study protool, the participant received the free and informed consent form via WhatsApp. Furthermore, a phone call was made one day before to perform the study protocol, primarily to exclude those with flu-like symptoms. Additionally, in our study, the following exclusion criteria were used: (i) absence of a positive diagnosis of COVID-19, (ii) use of NIV and/or oxygen therapy prior to inclusion in the study, and (iii) presence of flu-like symptoms during or prior to data collection, as described before.

**Figure 2:**
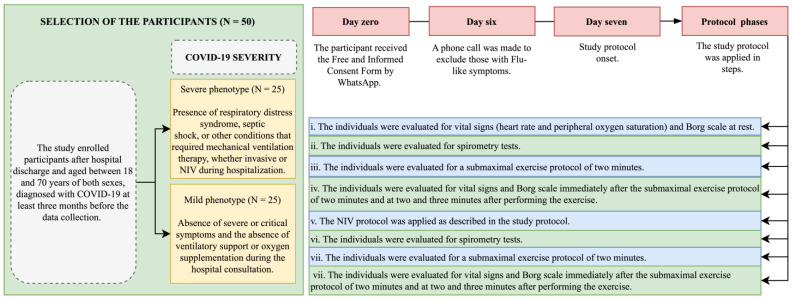
Study protocol. COVID-19, coronavirus disease (COVID-19); N, number of individuals (participants); NIV, non-invasive ventilation support.

Individuals were initially evaluated for vital signs (heart rate and peripheral oxygen saturation), the Borg scale (sensation of dyspnea), and the submaximal exercise protocol of two minutes of the step test before and after NIV. The vital signs and Borg scale were also measured for two minutes and three minutes of rest after the submaximal exercise protocol of two minutes. The lung function was only evaluated once before and after the submaximal exercise protocol of two minutes. None of the procedures caused immediate harm to the participants. The complete study protocol is presented in Figure 1 and Figure 2.

### 2.3. Submaximal Exercise Protocol of Two-Minute Step Test

The protocol was carried out with a portable step (kikos^®^, São Paulo 01234-001 São Paulo, Brazil), set at 19.5 cm for all participants, with the following dimensions: 66 cm wide and 37.5 cm long. The researchers demonstrated the procedures as part of the training. The exercise test lasted two minutes and the speed was adjusted according to the tolerance of each participant without jumps (submaximal exercise protocol). The test started with both feet on the ground, in front of the step. After the verbal command, the test was accompanied by verbal encouragement every 30 s [32,33,34,35]. The submaximal exercise protocol was implemented, always considering the safety of the participants. During the two-minute submaximal exercise protocol, the number of steps climbed was measured. In addition, as described below, participants were constantly monitored for vital signs.

### 2.4. Spirometry Test Protocol

The spirometry test was performed by a single professional using a portable spirometer model CPFS/D-USB^TM^ (MGC Diagnostics Co., Saint Paul, MN, USA) and using Breeze PF software version 3.8 B for Windows 95/98/NT (MGC Diagnostics Co., Saint Paul, MN, USA). The results presented are in accordance with the recommendations of the European Respiratory Society (ERS) and the American Thoracic Society (ATS) [35]. Each participant was asked to perform a vigorous and prolonged expiratory maneuver to achieve the reproducibility criterion of the forced vital capacity (FVC) maneuver. The spirometer equipment was calibrated immediately before each exam and the spirometry parameters evaluated in the study were as follows: (i) forced expiratory volume in the first second of the FVC (FEV1), (ii) FVC, (iii) ratio between FEV1 and FVC (FEV1/FVC), (iv) forced expiratory flow between 25% and 75% of the FVC (FEF25–75%), (v) forced expiratory flow at 25% of the FVC (FEF25%), and (vi) forced expiratory flow at 75% of the FVC (FEF75%). The values were described in predicted values, according to the literature [36].

### 2.5. Clinical Signs and Symptoms and Measurements on the Borg Scale

In this study, vital signs were evaluated before, during, and after the step test, with heart rate and peripheral oxygen saturation being evaluated. Markers were measured using a DellaMEDTM finger pulse oximeter (Itajai, Santa Catarina, Brazil), MD300C1 Premium model.

The modified Borg scale was used to evaluate subjective perception of exertion (sensation of dyspnea) using a laminated and printed visual table [37]. Participants were instructed to consider the degree of perceived subjective effort ranging from zero (no dyspnea) to ten (exhausting dyspnea). In this context, the higher the score, the greater the physical fatigue described by the participant. The Borg scale (sensation of dyspnea) was applied at rest (before), immediately after the submaximal exercise protocol (step test), and in the second and third minutes of rest after performing the step test.

### 2.6. NIV Protocol

The NIV was used through the BMC GII T-30T device BiPAP (bilevel positive airway pressure) medical machine (BMC Medical Co, Haidian, 100036 Beijing, China) in ventilation mode by applying continuous positive airway pressure of 6 cmH_2_O, and through BMC F5 face mask (BMC Medical Co, Haidian, 100036 Beijing, China), with face size and position suitable for each individual, for 30 min, only once, after collecting baseline data, spirometry, and two-minute step test data [33,38]. It was not necessary to use additional oxygen. Participants were instructed to breathe through their nose during NIV, spontaneously, each according to their respiratory demand. For all individuals, the same pressure and time values were used, regardless of age, sex, and severity phenotype of COVID-19 during active infection.

### 2.7. Statistical Analysis

Statistical analysis was performed using the Statistical Package for Social Sciences (IBM SPSS Statistics for Macintosh, Version 28.0. Armonk, NY, USA: IBM Corp.) with a significance level of 5%. The categorical data are presented as absolute frequency (N) and relative frequency (%) and the numerical data are presented as median (percentile 25% and percentile 75%) for tables and median and 95% confidence intervals for figures.

The normality of numerical data was assessed using the following techniques: (i) analysis of descriptive measures for central tendency, (ii) graphical method (normal Q-Q plot, Q-Q plot without trend, and boxplot), and (iii) statistical test methods (normality tests): Kolmogorov–Smirnov and Shapiro–Wilk tests.

To compare the biomarker values between the severity groups (severe and mild phenotypes of COVID-19), the Mann–Whitney test was used for independent samples, and to compare the marker values before and after NIV, the Wilcoxon test for paired samples and the Friedman one-way test for variance analysis for repeated measures were applied. In addition, to compare the distribution among markers set as categorical, the Chi-square test or the Fisher exact test was used according to the data distribution.

Figures were built using GraphPad Prism version 10.2.3 for Mac (http://www.graphpad.com, GraphPad Software, San Diego, CA, USA) accessed on 11 December 2024.

## 3. Results

This study enrolled 50 participants who contracted SARS-CoV-2 infection at least three months prior to the study protocol. Participants were divided into two groups of 25 individuals according to the severity phenotype of COVID-19 during active infection. Participants in the severe COVID-19 phenotype were older than the mild phenotype (*p*-value = 0.008) and had more comorbidities (*p*-value = 0.045) (Table 1).

Female participants were more common among those classified as mild (*p*-value = 0.038) (Table 1). White race was the most common between both COVID-19 severity phenotype groups without statistical differences and the vaccination status against COVID-19 at the time of hospitalization showed an equal distribution between the groups with 100% vaccination coverage for mild COVID-19 individuals and 96% coverage among those classified as severe phenotype. Among the participants who showed the severe disease phenotype, 60% reported pulmonary involvement during active infection (Table 1).

Participants in the severe phenotype group stayed 7 days in the hospital and 4 days with NIV (Table 1). Among the participants characterized as severe, four serious events (adverse events) were observed during active SARS-CoV-2 infection, with one case of pneumothorax that was drained and three cases of pneumomediastinum that were not drained.

All participants had a similar period between hospital discharge and then enrollment in the study protocol to reduce variability among individuals because fibrotic changes following COVID-19 can be an element of ambiguity in imaging status and, consequently, lung function and tolerance to exercise.

### 3.1. Spirometry Markers and Submaximal Exercise Protocol of a Two-Minute Step Test

**Figure 3:**
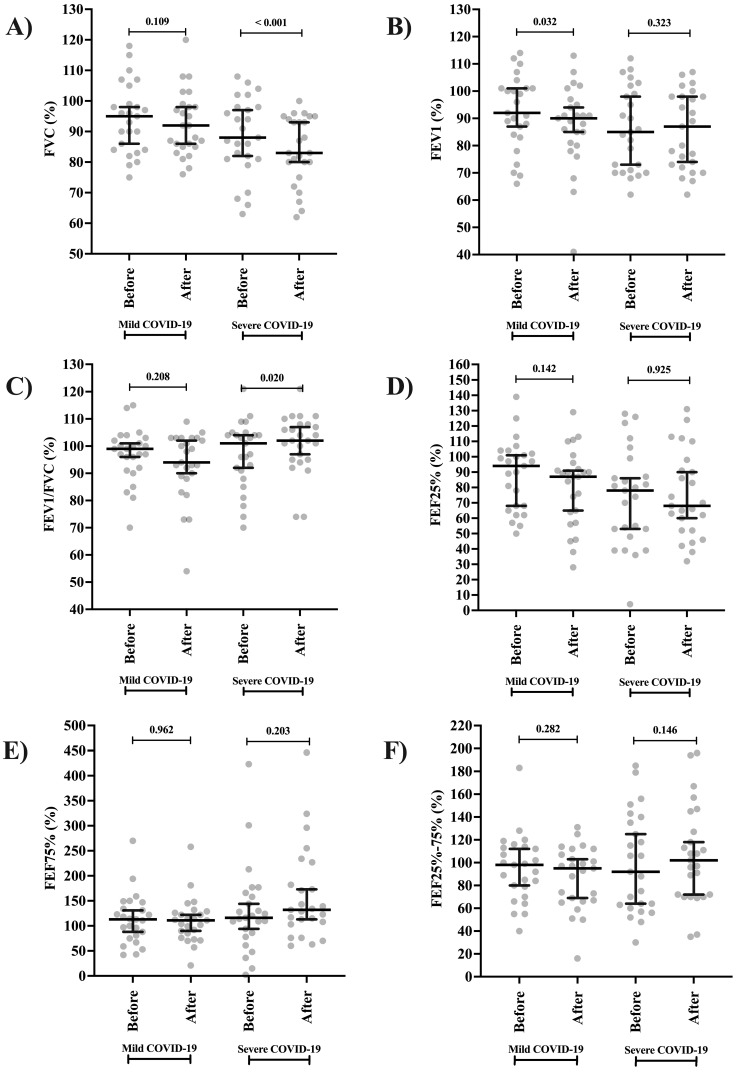
Lung function by spirometry in individuals affected by severe acute respiratory syndrome coronavirus 2 (SARS-CoV-2) according to the severity of the phenotype during active infection [coronavirus disease (COVID-19) phenotype] before and after the intervention (non-invasive ventilation support, NIV). (**A**) Forced vital capacity (FVC); (**B**) forced expiratory volume in the first one second to the forced vital capacity (FEV1); (**C**) ratio between FEV1 and FVC (FEV1/FVC); (**D**) forced expiratory flow at 25% (FEF25%); (**E**) forced expiratory flow at 75% (FEF75%); and (**F**) forced expiratory flow between 25% and 75% (FEF25–75%). Individuals were grouped according to the severity of COVID-19 into two groups: severe phenotype is the presence of respiratory distress syndrome, septic shock, or other conditions that required mechanical ventilation therapy, whether invasive or NIV during hospitalization, and mild phenotype is the absence of severe or critical symptoms and absence of ventilatory support or oxygen supplementation during hospital consultation. Statistical analysis was performed using the Wilcoxon signed-rank test for paired samples. An alpha error of 0.05 was adopted in all statistical analyses. %, percentage.

The spirometry markers and the comparison between groups and periods (before and after NIV) are presented in Table 2 and Figure 3A–F. Participants in the severe phenotype groups had a lower FVC after receiving NIV (Figure 3A). On the contrary, this group of individuals showed improvement in the FEV1/FVC (Figure 3C) and FEF75% (Figure 3E). Among those classified as mild phenotype, low FEV1 values were observed after receiving NIV (Figure 3B). In the comparison between the groups, those classified as severe phenotype presented lower FVC values and higher FEV1/FVC and FEF75% values than those classified as mild phenotype (Table 2). Furthermore, no differences occurred for FEF25% (Figure 3D) and FEF25–75% (Figure 3F) between the groups (COVID-19 phenotype) and periods (before and after NIV).

**Figure 4:**
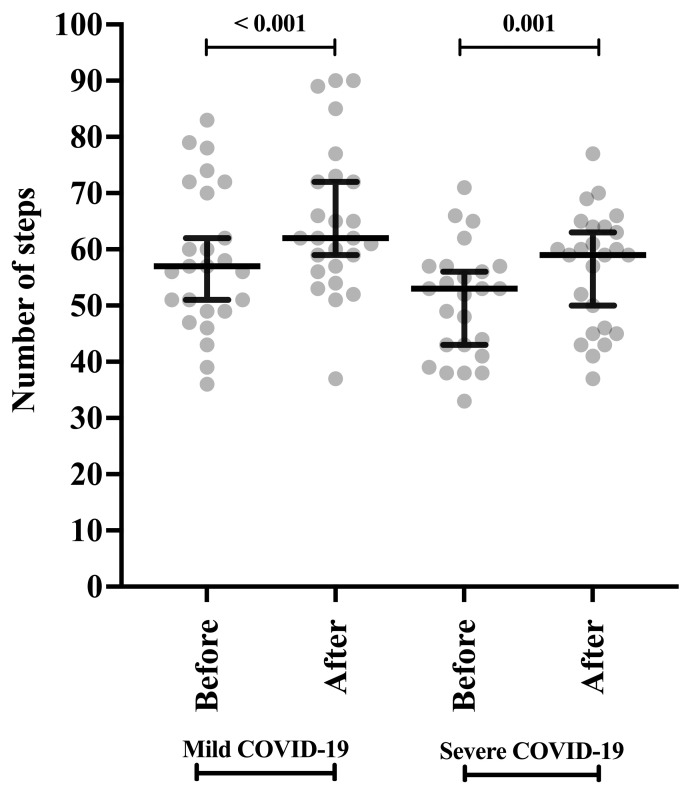
Submaximal exercise protocol of two-minute step test in individuals affected by severe acute respiratory syndrome coronavirus 2 (SARS-CoV-2) according to the severity of the phenotype [coronavirus disease (COVID-19) phenotype] during active infection before and after receiving the intervention (non-invasive ventilation support, NIV). Individuals were grouped according to the severity of COVID-19 into two groups: severe phenotype is the presence of respiratory distress syndrome, septic shock, or other conditions that required mechanical ventilation therapy, whether invasive or NIV during hospitalization, and mild phenotype is the absence of severe or critical symptoms and absence of ventilatory support or oxygen supplementation during hospital consultation. Statistical analysis was performed using the Wilcoxon signed-rank test for paired samples. An alpha error of 0.05 was adopted in all statistical analyses.

Both groups of participants [coronavirus disease (COVID-19) phenotype] presented a higher number of steps after receiving NIV: [(mild phenotype) 57 steps vs. 62 steps (*p*-value < 0.001) and (severe phenotype) 53 steps vs. 59 steps (*p*-value < 0.001)] (Table 2; Figure 4). Furthermore, the participants with a mild phenotype were able to walk more steps before (57 steps vs. 53 steps; *p*-value = 0.042) and after (62 steps vs. 59 steps; *p*-value = 0.042) receiving NIV (*p*-value = 0.042) than those with severe phenotype (Table 2).

### 3.2. Vital Signs and Sensation of Dyspnea (Borg Scale)

**Figure 5:**
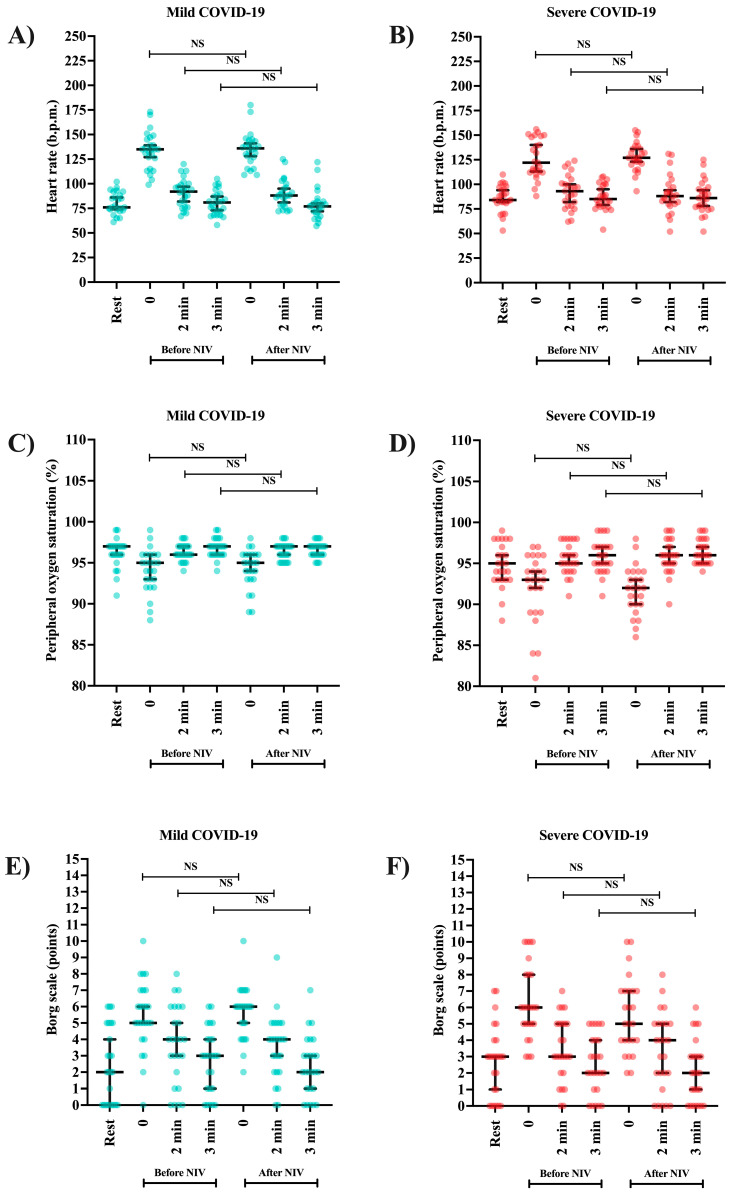
Association of heart frequency, peripheral oxygen saturation, and Borg scale for individuals affected by severe acute respiratory syndrome coronavirus 2 (SARS-CoV-2) according to the severity of the phenotype [coronavirus disease (COVID-19) phenotype] during active infection before and after the intervention (non-invasive ventilation support, NIV). (**A**) Heart rate—mild COVID-19 phenotype; (**B**) Heart rate—severe COVID-19 phenotype; (**C**) Peripheral oxygen saturation—mild COVID-19 phenotype; (D) Peripheral oxygen saturation—severe COVID-19 phenotype; (**E**) Borg scale—mild COVID-19 phenotype; and (**F**) Borg scale—severe COVID-19 phenotype. Statistical analysis was performed using the Wilcoxon signed-rank test of the paired sample. An alpha error of 0.05 was adopted in all statistical analyses. NS, no significance; b.p.m, beats per minute; NIV, non-invasive ventilation; %, percentage; min, minutes.

Descriptions of vital signs and the Borg scale are presented in Table 3 and Figure 5A–F. In both groups (mild and severe COVID-19 phenotypes) and periods (before and after receiving NIV), heart rate and the Borg scale increase from rest to the start of a two-minute submaximal exercise protocol, with a decrease in their values by two and three minutes at rest after performing the test (Table 3). On the contrary, for peripheral oxygen saturation, the values decrease from rest to the beginning of the submaximal exercise protocol of two minutes, with an increase in their values by two and three minutes at rest after the test (Table 3). Interestingly, in the paired analysis for the same severity group by periods, none of the markers presented significant statistical differences (Figure 5).

The heart rate values were higher among those classified as severe phenotype compared with those classified as mild phenotype for both periods—before receiving NIV and after receiving NIV at three minutes after the submaximal exercise protocol of two minutes. The peripheral oxygen saturation was lower among those classified as severe phenotype compared with those classified as mild phenotype after receiving NIV immediately after the submaximal exercise protocol of two minutes and at three minutes after the submaximal exercise protocol of two minutes (Table 3).

## 4. Discussion

Individuals who have been affected by COVID-19 may experience sequelae described as post-COVID-19 syndrome [3]. In these cases, it becomes important to monitor individuals to deal with clinical phenotypes arising mainly from SARS-CoV-2 infection in critical cases requiring hospitalization, often with the mutual need for intubation. The evaluation of patients and their management must be multifactorial and with a concern associated with the condition with the highest degree of involvement. Among the aspects associated with post-COVID-19 syndrome, there is great concern, mainly related to lung disease [3]. In this regard, different tools can be used to improve, mainly, the quality of life of patients affected by sequelae. In this context, NIV emerges as a tool that can improve the clinical condition of the respiratory tract. Taking into account this assumption, the present study evaluates the use of NIV in patients after COVID-19 diagnosis according to the degree of severity assessed during active SARS-CoV-2 infection. Interestingly, in this study, the main findings were increased tolerance to the submaximal exercise test, modulation of lung function values, improvement in cardiac function and peripheral oxygen saturation, and reduction in the sensation of dyspnea.

In the present study, as described above, it was observed that NIV was capable of modulating different markers, including exercise tolerance and the sensation of dyspnea. In patients with heart failure, data similar to those described by us were observed, where NIV with the use of BiPAP showed beneficial effects on exercise tolerance and dyspnea [38]. Furthermore, as presented by us, the use of the tool was safe and well tolerated by the study participants and, in this group of patients, the tool was considered for inclusion in cardiac rehabilitation programs [38]. The implementation of walking distance and chronotropic reserve in patients with chronic respiratory insufficiency also occurred in the use of continuous positive airway pressure (CPAP) for 30 min with progression of 4 to 6 cmH_2_O, as described in a double-blind, randomized, crossover, and placebo-controlled protocol, with 12 participants [39].

In the literature, high-intensity physical exercise is described to be associated with redistribution of blood flow from peripheral muscles to ventilatory muscles [26]. Change in muscle blood flow occurs due to the ~30% increase in relative cardiac output and overload of ventilatory muscles. As a consequence of this process, early fatigue will be induced due to the decrease in the blood supply to the peripheral muscles [24,25,26]. In this context, since the application of CPAP and, possibly, BiPAP decreases left ventricular transmural pressure, improving cardiac output and reducing end-systolic volume, optimization of cardiac performance may have occurred by increasing tolerance to physical exercise in patients with chronic heart failure [33]. Therefore, our findings may be related to increased tolerance to physical exercise, given by optimizing oxygenation in peripheral muscles through redistribution of blood flow, even with BiPAP of 6 cmH_2_O.

Furthermore, the use of NIV during physical exercise is recognized as a resource to treat chronic obstructive pulmonary disease and congestive heart failure. NIV is capable of improving the distance covered during the six-minute walk test and, in the literature, the use of NIV was better than exercise alone in improving oxygen saturation and respiratory rate, promoting an impact on exercise performance and quality of life [40]. In this context, NIV is a treatment alternative to reverse the decompensation of congestive heart failure and improve oxygenation in patients with chronic obstructive pulmonary disease, providing positive changes in alveolar and intrathoracic pressure, as well as in the activity of pulmonary receptors, and it can promote an immediate gain in exercise capacity through greater oxygen capture at the cellular level [41].

In post-COVID-19, people have described a restrictive lung disease pattern related to pulmonary fibrosis [42]. Lung injuries during the acute phase of COVID-19 can promote changes in gas exchange and the mechanics of the respiratory system. Oxygen supplementation and ventilatory support are provided while the body waits for the body to respond to the imbalance imposed by SARS-CoV-2 infection and the cytokine storm [41,42,43]. Decreased lung compliance and increased lung inflammation are the main theories associated with the occurrence of pulmonary fibrosis in the chronic phase of COVID-19 [14,15].

NIV has been associated with physical training to care for patients with different diseases with chronic cardiorespiratory and pulmonary profiles. In this context, benefits have been provided, mainly in the perception of dyspnea, which is an important factor associated with quality of life. Our results did not corroborate these findings, since individuals, especially those with severe phenotype during active SARS-CoV-2 infection, have no alteration in their heart rate and peripheral oxygen saturation values after the use of NIV, raising the possibility that the reduction in the heart rate may have been linked to the reduction in inspiratory effort only in some special cases [42,43]. However, in this study, the group of participants classified as having severe phenotypes had a lower peripheral oxygen saturation value. The lower values described may be associated with greater pulmonary impairment during hospitalization and, concomitantly, the need for oxygen therapy and positive respiratory pressure to induce clinical recovery of the patient during the acute phase of the infection caused by SARS-CoV-2 [43]. Supplemental oxygen is routinely used to treat reduced blood oxygen saturation in COVID-19. However, prolonged exposure to hyperoxia can cause oxygen toxicity, due to an excessive increase in the levels of reactive oxygen species, which consequently can overload cellular antioxidant capacity [43]. Subsequently, hyperoxia causes oxidative cellular damage and elevated levels of aging biomarkers, such as telomere shortening and inflammation. In addition, the possibility of pulmonary fibrosis in severe cases is theorized, which inhibits gas exchange and lung expansion and causes lower peripheral oxygen saturation [15].

As previously described, the benefits observed in lung function resulting from CPAP and, possibly BiPAP, are due to the increase in intrathoracic pressure and, consequently, the reduction in left ventricular afterload due to the pressurization of the airways [43,44]. On the other hand, pressurization increases cardiac output and, thus, promotes improvements in lung volumes and capacities, increasing residual functional capacity and optimizing the opening of collapsed or hypoventilated alveoli with subsequent reduction in intrapulmonary shunt and improvement in oxygenation [44]. In this context, as observed in patients with cystic fibrosis, NIV is capable of improving lung mechanics by increasing airflow and gas exchange and reducing respiratory work and myocardial overload [44,45]. Furthermore, our study only evaluated lung function using the spirometry test. In this context, in future studies, measurements of the diffusing capacity of the lung for carbon monoxide can be used to increase the sensitivity and scope of the assessment by providing valuable insight into the efficiency of gas exchange in the lung, as demonstrated in the literature [45,46,47]. Among the findings associated with lung function, in our study, the FVC was lower and, consequently, the ratio between FEV1/FVC was higher after the use of NIV in participants with severe phenotype during active SARS-CoV-2 infection. The findings described are in line with the literature that describes the greater reduction in respiratory muscle strength, especially in the presence of a greater severity associated with COVID-19 [48,49].

It is now known that symptomatic patients after 3 months of COVID-19 infection had important clinical implications related to respiratory symptoms, such as decreased lung function, in subjective assessment of quality of life and in computed tomography. In these patients with respiratory sequelae, anti-S levels were found to be significantly associated with severe COVID-19 and carbon monoxide diffusing capacity corrected for hemoglobin below 80%, as well as bilateral thoracic anomalies on computed tomography [50].

Concerning the present study, the pressure used was 6 cmH_2_0, since the groups were heterogeneous, and higher-pressure values have been associated with increased mortality, probably due to increased hyperinflation of lung portions with better compliance [48,49]. Our protocol included the exercise modality with submaximal effort in the step test, which proved to be useful for evaluating cardiorespiratory function in both groups. The choice of exercise modality with submaximal effort was due to the difficulty in establishing a maximal exercise protocol in our group of individual participants. Furthermore, as described in the literature and carried out by us, step tests should preferably be carried out 3, 6, and 12 months after hospital discharge [14,15]. Furthermore, participants of this study were evaluated only three months after discharge from the hospital.

Importantly, from a physiological and pathological perspective, it must be recognized that improved functional parameters may be primarily due to physical effort facilitated by the step test. This effort can contribute to the ventilation of previously unventilated alveolar areas and to the regulation of the ventilation–perfusion ratio. In this context, other studies should be performed to evaluate this information and confirm our findings.

This study presented some important findings about the use of NIV in patients affected by COVID-19. However, caution is needed when interpreting the findings due to the limitations of the proposed study model, among which we highlight the inclusion of a convenience sample, variability in the time of inclusion after the COVID-19 phenotype, lack of epidemiological data and robust population clinicians prior to inclusion in the study, and inclusion of a limited number of tools to analyze response to NIV in study participants. The presence of a control group, COVID-19 survival without CPAP augmentation or healthy individuals who are put on the same exercise test with the use of CPAP, could significantly strengthen the current argument. In addition, this study evaluated the participants only after a single session of CPAP treatment. It did not evaluate the long-term effects or the sustainability of the observed benefits. Furthermore, despite the benefits described, there is still a need for further studies that focus on injuries caused by previously used pressures, as well as the pressures themselves being well defined in their choices. We believe in the need for continued studies to better understand the benefits caused by NIV on cardiorespiratory function in chronic diseases, as well as in patients with post-COVID-19 syndrome.

## 5. Conclusions

With the help of the two-minute submaximal exercise protocol in the step test, it was possible to identify that NIV is a tool that helps in the treatment of people who have had COVID-19 with lung complications, improving lung function immediately after its use, observed by improving the spirometry variables and increased exercise capacity observed by the increase in the number of steps.

## Figures and Tables

**Table 1:** Demographical and clinical characteristics of people affected by severe acute respiratory syndrome coronavirus 2 (SARS-CoV-2) according to the severity of the phenotype during active infection.

Marker	Data	Mild COVID-19 *	Severe COVID-19 *	*p*-Value
Sex	Female	20 (80%)	12 (48%)	**0.038 ^a^**
	Male	5 (20%)	13 (52%)	
Age during hospitalization (years)		34 (29.00 to 40.50)	46 (34 to 54)	**0.008 ^c^**
Race	White people	22 (88%)	23 (92%)	1.000 ^b^
	Mixed people	3 (12%)	2 (8%)	
Vaccine against coronavirus disease (COVID-19)	No information	0	1 (4%)	0.235 ^b^
	1 dose	0	2 (8%)	
	2 doses	25 (100%)	22 (88%)	
Comorbidities	Absent (none)	18 (72%)	10 (40%)	**0.045 ^b^**
	Present (≥1)	7 (28%)	15 (60%)	
Pulmonary involvement during active infection	No information	NA	2 (8%)	NA
	>50%	NA	16 (60%)	
	≤50%	NA	7 (32%)	
Hospitalization (days)		NA	7.00 (5.00 to 12.00)	NA
Non-invasive ventilation (NIV, days)		NA	4.00 (2.50 to 7.00)	NA
NIV interface during hospitalization	NIV	NA	16 (60%)	NA
	NIV + others	NA	10 (40%)	

NA, not applicable. The categorical data are presented as absolute frequency (N) and relative frequency (%); the numerical data are presented as median (percentile 25% and percentile 75%). ^a^ The Chi-square test was applied; ^b^ the Fisher exact test was applied; ^c^ the Mann–Whitney test for independent samples was applied. An alpha error of 0.05 was adopted in all statistical analyses. Significant *p*-values are marked with the bold type. * The individuals were grouped according to COVID-19 severity into two groups: severe phenotype is the presence of respiratory distress syndrome, septic shock, or other conditions that required mechanical ventilation therapy, whether invasive or NIV during hospitalization, and mild phenotype is the absence of severe or critical symptoms and the absence of ventilatory support or oxygen supplementation during the hospital consultation.

**Table 2:** Association of lung function through spirometry and step test variables for individuals affected by severe acute respiratory syndrome coronavirus 2 (SARS-CoV-2) according to the severity of the phenotype during active infection [coronavirus disease (COVID-19) phenotype] before and after the intervention (non-invasive ventilation support, NIV).

Biomarkers	Groups *	Before Receiving NIV	After Receiving NIV	*p*-Value ^a^
FVC	Mild	95.00 (84.00 to 102.00)	92.00 (83.50 to 101.00)	0.109
	Severe	88.00 (81.00 to 98.00)	83.00 (77.50 to 94.50)	**<0.001**
	*p*-value ^b^	0.187	**0.021**	
FEV1	Mild	92.00 (85.00 to 98.50)	90.00 (80.50 to 96.00)	**0.032**
	Severe	85.00 (70.50 to 100.50)	87.00 (72.50 to 98.00)	0.323
	*p*-value ^b^	0.171	0.580	
FEV1/FVC	Mild	99.00 (91.50 to 101.50)	94.00 (88.67 to 103.00)	0.208
	Severe	101.20 (89.50 to 104.50)	102.00 (95.00 to 108.00)	**0.020**
	*p*-value ^b^	0.634	**0.008**	
FEF25%	Mild	94.00 (66.50 to 103.00)	87.00 (60.50 to 94.00)	0.142
	Severe	78.00 (50.50 to 93.00)	68.00 (52.00 to 94.61)	0.925
	*p*-value ^b^	0.064	0.382	
FEF75%	Mild	113.00 (78.00 to 140.50)	111.00 (80.50 to 127.50)	0.968
	Severe	116.00 (82.00 to 162.00)	132.20 (105.50 to 204.50)	**0.023**
	*p*-value ^b^	0.628	**0.029**	
FEF25–75%	Mild	98.00 (74.50 to 113.50)	95.00 (66.50 to 109.50)	0.282
	Severe	92.00 (61.50 to 137.50)	102.00 (71.00 to 136.00)	0.146
	*p*-value ^b^	0.900	0.130	
Number of steps	Mild	57.00 (49.00 to 71.00)	62.00 (56.50 to 72.50)	**<0.001**
	Severe	53.00 (42.00 to 57.00)	59.00 (45.50 to 64.05)	**<0.001**
	*p*-value ^b^	**0.042**	**0.042**	

The numerical data are presented as median (percentile 25% and percentile 75%). ^a^ The Wilcoxon signed-rank test for paired samples was applied. ^b^ The Mann–Whitney test for independent samples was applied. An alpha error of 0.05 was adopted in all statistical analyses. Significant *p*-values are marked with the bold type. FCV, forced vital capacity; FEV1, forced expiratory volume in the first one second to the forced vital capacity; FEF25%, forced expiratory flow at 25%; FEF75%, forced expiratory flow at 75%; FEF25–75%, forced expiratory flow between 25% and 75%. * Individuals were grouped according to COVID-19 severity into two groups: severe phenotype is the presence of respiratory distress syndrome, septic shock, or other conditions that required mechanical ventilation therapy, whether invasive or NIV during hospitalization, and mild phenotype is the absence of severe or critical symptoms and absence of ventilatory support or oxygen supplementation during hospital consultation.

**Table 3:** Association of vital signs (heart frequency and peripheral oxygen saturation) and sensation of dyspnea (Borg scale) for individuals affected by severe acute respiratory syndrome coronavirus 2 (SARS-CoV-2) according to the severity of the phenotype during active infection [coronavirus disease (COVID-19) phenotype] before and after the intervention (non-invasive ventilation support, NIV).

Biomarkers	Groups *	Rest	Before Receiving NIV			After Receiving NIV			*p*-Value ^a^
			0	2 min	3 min	Post	2 min	3 min	
Heart frequency	Mild	76 (73.5 to 90.0)	135 (120.5 to 146.0)	92 (78.00 to 99.50)	81 (70.0 to 89.0)	136 (125.0 to 143.5)	88 (78.5 to 98.0)	77 (70.0 to 82.0)	**<0.001**
	Severe	84 (81.0 to 96.0)	122 (112.0 to 148.5)	93 (78.0 to 103.0)	85 (77.5 to 99.5)	127 (120.5 to 137.5)	88 (80.0 to 98.5)	86 (76.0 to 96.0)	**<0.001**
	*p*-value ^b^	0.114	0.277	0.705	**0.048**	0.130	0.938	**0.035**	
SpO_2_	Mild	97 (96.0 to 97.0)	95 (92.5 to 96.0)	97 (95.5 to 97.0)	97 (96.0 to 97.0)	95 (93.0 to 95.0)	96 (96.0 to 97.0)	97 (96.0 to 97.5)	**<0.001**
	Severe	95.00 (93.0 to 97.5)	93.00 (89.0 to 95.5)	96.00 (95.0 to 97.5)	96 (95.0 to 98.0)	92 (90.0 to 93.93)	95 (94.0 to 97.4)	96 (94.5 to 97.0)	**<0.001**
	*p*-value ^b^	0.060	0.105	0.272	0.676	**<0.001**	0.079	**0.046**	
Borg scale	Mild	2 (0.0 to 5.0)	5 (4.5 to 7.0)	4 (1.5 to 6.0)	3 (1.0 to 4.0)	6 (4.5 to 6.7)	4 (2.0 to 5.0)	2 (1.0 to 3.5)	**<0.001**
	Severe	3 (0.50 to 4.0)	6 (5.0 to 8.0)	3 (2.0 to 5.0)	2 (1.0 to 4.0)	5 (4.00 to 7.0)	3.75 (1.5 to 7.0)	2.0 (0.0 to 3.0)	**<0.001**
	*p*-value ^b^	0.533	0.283	0.624	0.738	0.666	0.769	0.602	

Numerical data are presented as median (percentile 25% and percentile 75%). ^a^ The Friedman one-way repeated measure analysis of variance by rank test was applied. ^b^ The Mann–Whitney test for independent samples was applied. An alpha error of 0.05 was adopted in all statistical analyses. Significant *p*-values are marked with the bold type. * Individuals were grouped according to the severity of COVID-19 into two groups: severe phenotype is the presence of respiratory distress syndrome, septic shock, or other conditions that required mechanical ventilation therapy, whether invasive or NIV during hospitalization, and mild phenotype is the absence of severe or critical symptoms and absence of ventilatory support or oxygen supplementation during hospital consultation. SpO_2_, peripheral oxygen saturation.

## Data Availability

The original contributions presented in this study are included in the article. Further inquiries can be directed to the corresponding author.
